# Perceptions of minimum age at marriage laws and their enforcement: qualitative evidence from Malawi

**DOI:** 10.1186/s12889-021-11434-z

**Published:** 2021-07-08

**Authors:** Andrea J. Melnikas, Nancy Mulauzi, James Mkandawire, Sajeda Amin

**Affiliations:** 1 Population Council, 1 Dag Hammarskjold Plaza, 3rd floor, New York, NY 10017 USA; 2 Invest in Knowledge, Plot Number 43, Chirunga Road, Box, 506 Zomba, Malawi

**Keywords:** Child marriage, Age at marriage laws, Qualitative

## Abstract

**Background:**

Child marriage in Malawi is a significant problem with 42.1% of women 20–24 married by age 18. In 2017 the Malawi government formalized legislation to make marriage under age 18 illegal; violators are subject to fines. While leveraging laws to reduce child marriage is common, the enactment of laws and their enforcement has led to some novel practices. One such practice observed in Malawi is marriage withdrawal, where the community intervenes when a child marriage has taken place to force the girl to return to her natal home.

**Methods:**

This paper is a qualitative analysis of perceptions regarding marriage withdrawal. We conducted focus group discussions and in-depth interviews with married and unmarried adolescents, parents of adolescents, and key community members in Mangochi and Nkhata Bay. Data were collected as part of an evaluation of the More Than Brides Alliance program aimed at delaying marriage and improving access to sexual and reproductive health services in Malawi.

**Results:**

The knowledge that violation of marriage laws entails substantial fines is widespread and marriage withdrawals are seen by some respondents as a way of enforcing the spirit of child marriage laws while avoiding fines. Some respondents suggest that enforcement of marriage laws has an unintended effect of driving marriages underground. One important disconnect between the laws and the realities of child marriage practices in these communities is that the law holds parents responsible for the marriage and for preventing it, while parents do not necessarily exercise control, particularly when the marriage is precipitated by pregnancy. While parents and other adults view withdrawals as an acceptable resolution of a problematic child marriage, girls noted many drawbacks for withdrawn girls such as stigma and limited education and livelihood opportunities once withdrawn.

**Conclusions:**

Our exploration of perceptions about marriage laws suggest that the imposition of fines may have some unintended consequences, both driving the practice underground and encouraging practices to evade fines, and may be associated with unintended consequences for adolescent girls. Programs to address child marriage should include other approaches that address more distal drivers including poverty and lack of alternatives to child marriage.

**Trial registration:**

This work is part of an RCT registered August 4, 2016 in the AEA RCT registry identified as: AEARCTR-0001463. See: https://www.socialscienceregistry.org/trials/1463

**Supplementary Information:**

The online version contains supplementary material available at 10.1186/s12889-021-11434-z.

## Background

### Child marriage and adolescent childbearing in Malawi

Despite a small increase in mean age at first marriage for women in Malawi (from 17.8 years in 1992 to 18.2 in 2015/6 among women 25–49), child marriage, or marriage before the age of 18, remains a significant issue. Survey data suggest that 42.1% of all women age 20–24 report being married by age 18, down from nearly half in 2010 (49.6%) but still higher than neighboring Zambia (29.0%) and Zimbabwe (32.4%). Baseline studies conducted as part of this project found that 31.0% of girls 15–19 were ever married, with a median age at marriage of 16.4 years in Mangochi and 17.2 years in Nkhata Bay [1]. As of 2015, Malawi had the ninth highest rate of child marriage in the world [2].

According to the Demographic and Heath Surveys (DHS) sexual initiation is common in adolescence in Malawi where 59.7% of women 20–24 report having had sex by age 18, and 16.6% by age 15 [2]. We found that about 1 in 3 ever married girls in Nkhata Bay (35.7%) reported being forced into marriage by pregnancy. This represents a significant proportion of girls who report pregnancy influencing marriage timing [1] and represents a difference in the antecedents of child marriage in Malawi compared to other high child marriage countries. For example, in Niger, 76% of women 20–24 report being married before age 18 and pregnancy almost always follows marriage: in an adolescent survey in 2017 we found that almost no reported pregnancies (0.6%) were to unmarried girls [3, 4].

### Minimum age at marriage laws

Despite global gains in reducing child marriage, the practice remains a significant issue. Research on child marriage finds that the practice increases the risk of intimate partner violence, reduces girls’ likelihood of being enrolled in school, and is associated with early childbearing and accompanying risks [5–7]. Internationally, many bodies have addressed the practice: the Universal Declaration of Human Rights states that those entering into marriage must do so freely and be at full age and the Convention on the Elimination of All Forms of Discrimination Against Women declares child marriage illegal [8, 9]. In sub-Saharan Africa, the United Nations Convention on the Rights of the Child (1989) and the 1990 African Charter on the Rights and Welfare of the Child (ACRWC) protect the rights of children [10]. As Maswikwa and colleagues (2015) note, the ACRWC compelled countries to take action to end child marriage and set a minimum age for marriage. In response to that, many countries have strengthened laws against marriage under age 18 [10]. With global emphasis on the rights of children and how child marriage infringes on those rights, advocacy groups such as Girls Not Brides have emerged to support and carry forward efforts to strengthen and raise awareness of laws as a tool to reduce child marriage [11].

Although the ACRWC occurred in 1990 and many other African countries addressed minimum age at marriage laws with varying degrees of flexibility [12], Malawi did not outlaw child marriage until very recently. Following the passage of the Marriage, Divorce and Family Relations Bill in 2015 which was signed into law by the president in 2017, child marriage was effectively banned. This bill made marriage before the age of 18 for both boys and girls illegal and included a 10-year imprisonment penalty for those violating the law [13, 14].

### Marriage laws in Malawi

When the government passed the Marriage, Divorce, and Family Relations Bill (2015), it assigned the same legal status to all four types of marriage practiced in Malawi: customary, religious, civil, and marriage by permanent cohabitation [14]. Prior to that, different types of marriages were also subject to alternative, and sometimes conflicting, sets of laws. Under customary law, parties must have reached puberty, the girl's parents must consent to the marriage, *lobola* must be paid (patrilineal), the girl or woman must be unmarried, and *chinkhoswe*, or an engagement ceremony, must occur (matrilineal) [15]. The Marriage, Divorce, and Family Relations Bill outlaws child marriage across all types of practices. Communities may also set specific bylaws around child marriage; these bylaws may also signal community commitment to specific causes [16].

### Enforcement of laws in Malawi

Although the 2015 Marriage, Divorce, and Family Relations Bills effectively banned child marriage, in practice child marriages still occur in some parts of Malawi [1, 2]. The most recent DHS, conducted before changes to the laws governing child marriage were implemented, shows a slight increase in marriage among women 15–19, with 21.5% of those 15–19 report being married or in union (DHS 2015/6) compared to 19.5% of those 15–19 in 2010. This followed a decline from 29.8% of those 15–19 reporting being married in 2004 [17].

To address child marriage many local organizations leverage the illegality of child marriage in program strategies to reduce child marriage. Strategies include providing education about the laws governing child marriage as well as enforcing laws (in cooperation with law enforcement and community leaders, in some cases) through practices such as marriage withdrawal [13, 18, 19]. Marriage withdrawal is the practice of removing a girl from her marital home, usually working with a local child protection committee or local government leader [19]. The ‘withdrawn’ girl is then returned to her natal home and often given support to return to school. Although there is limited research on marriage withdrawal in Malawi, multiple news articles report on this strategy as an approach to reducing child marriage [19].

### Marriage dissolution in Malawi

Although it is not clear how common marriage withdrawal is (there is no data on marriage withdrawals overall) both marriage dissolution and single motherhood are not uncommon [20–23]. In Malawi 10% of women are currently divorced, according to the 2015/6 DHS [2]. Higher divorce rates have been found in younger cohorts: the Malawi Schooling and Adolescent Survey found that only 58% of marriages were intact after 5 years [24]. Higher divorce among younger groups may explain why marriage withdrawal is seen as an acceptable strategy to deal with child marriage.

### Research on effect of laws on child marriage

Much of the previous research on child marriage and its correlates has focused on individual- and community- level factors to both understand and address the practice, and less on the legal environment and its effects on child marriage practices [9, 25–27] though as others have noted interventions that strengthen legal frameworks and leverage laws are not uncommon [12]. Previous research using data from countries in Sub-Saharan Africa, including Malawi, found that countries with consistent laws that set the minimum age of marriage at 18 had higher median ages at marriage [10]. At the time that article was published (2015), Malawi was considered to have inconsistent laws since the minimum age at marriage was legally under age 18; laws have since been passed to set the minimum age to 18. Maswikwa and colleagues suggest that setting consistent laws over age 18 may protect girls from exploitation. However, recent research suggests that despite consistent laws that set the minimum age at 18 more than 7.5 million girls globally marry illegally each year [28].

A review of interventions to address child marriage (2016) included a review of programs that focused on fostering an enabling legal and policy environment [29]. Three programs focused on the legal and policy environment as a primary strategy: these ‘activist programs’, as the authors refer to them, were less rigorously evaluated and less effective, according to this review. The authors suggest that legal and policy efforts should be complemented with other programmatic approaches to maximize impact on reducing child marriage [29], similar to suggestions by Wodon and colleagues (2017) [28]. One such study that focused exclusively on policy change examined the enforcement of minimum age at marriage laws in Indonesia and found that the law did not appear to have had much direct effect. However, the authors suggest that the law may instead have had an indirect effect by putting value on women’s autonomy and choice and providing possibilities for alternative roles for women [30].

Programs that target strengthening laws or work to raise awareness of laws must grapple with how these laws function to reduce child marriage: to use an analogy from health, laws may be either preventive or curative. In this analogy, a preventive approach is when laws act as a deterrent, where those who may consider allowing a girl under 18 to get married are dissuaded from that action due to the threat of punishment. Similarly, the articulation of a law may act as a deterrent purely because of its moral authority. A curative approach is when the laws are used to enforce desired norms around child marriage, by using the weight of the law to break up known child marriages by taking girls back, or withdrawing them, from marriages. Another example of a curative approach is having a child marriage declared invalid and voided. It is also important to note that when marriages are broken up in the name of the law, child marriage rates may not immediately decline as measurement of child marriage typically relies on women over age 18 reporting on whether they were ever married by age 18 [31]. However, fewer girls will remain in these marriages which may be seen as advantageous by these communities [19].

We sought to understand how knowledge and enforcement of minimum age at marriage laws influence perception of child marriage and timing of marriage in Malawi. Using qualitative data from interviews with married and unmarried adolescent girls, parents, and key community individuals, we explore knowledge of child marriage laws and how the law influences behaviors, including real and perceived repercussions of strategies such as marriage withdrawal as a response to these laws.

## Methods

This research was nested within a larger research study examining the impact of the More Than Brides Alliance (MTBA) intervention.^1^ Qualitative data were collected as part of this study in April 2018 and data collection focused on exploring adolescent social life, the marital process, and education and livelihoods in-depth, following on findings from the baseline survey [1]. This analysis focuses on the minimum age at marriage law and its influence on behaviors. Results from other domains are part of another manuscript in development.

### Study sites

Data collection took place in six villages in Northern Malawi (Nkhata Bay) and Southern Malawi (Mangochi). Study sites were selected using baseline data (2016) from MTBA [1] to identify enumeration areas that had a high proportion of ever-married adolescents to support focus groups with married girls: the proportion of girls 12–19 ever married in selected enumeration areas ranged from 18.2 to 40.9%. Within these selected areas with high child marriage prevalence, sites were chosen to achieve some diversity in ethnicity and religious affiliation. Nkhata Bay study sites are predominantly Christian (98.3%) with about 70% reporting Tonga ethnicity. Mangochi study areas are predominantly Muslim (95.5%) and Yao ethnicity (93.3%) [1]. Sites were selected from both comparison and intervention areas that were part of the MTBA program.

We recruited individuals who met selection criteria for participation in one of four data collection activities:

- Focus group discussions (FGDs) with parents of adolescent girls ages 12–19 with mother and father groups conducted separately;
- FGDs with adolescent girls ages 12–19 themselves, including unmarried, engaged, married girls and those who had a child;
- In-depth interviews (IDIs) with married girls ages 15–19 and unmarried girls ages 12–19; and
- IDIs with key informants, including local government officials, NGO workers and teachers to understand individual perspectives from those who may be part of local child protection committees.^2^

Data collection was carried out by an experienced social science research group, Invest in Knowledge (IKI) based in Zomba, Malawi. Six research assistants with experience in qualitative data collection were trained over a period of 4 days in April 2018. Interviewer training covered qualitative methods and limitations, a review of the study instruments, methods for accurate translation and transcription of data, and processes for obtaining informed consent/assent from study participants.

More detail on data collection activities is provided in Table 1. We used criterion sampling to create homogenous samples [32] of individuals according to age, marital status or parental status; we therefore over-represent married adolescents compared to our baseline sample. Parental permission and informed consent procedures were followed prior to including individuals in the research.

**Table 1:** Overview of Data Collection

	N
**In-depth interviews**	
Adolescents (married and unmarried)	21
Key informants (adults in community)	18
***Total in-depth interviews***	***39***
**Focus group discussions**	
Adolescents (married and unmarried)	124
Fathers	22
Mothers	25
***Total focus group participants (groups)***	***171 (20)***
***Total transcripts***	***59***

In the study areas in this research, we draw from both patrilineal and matrilineal areas and use the term *marriage* to refer to a recognized union. We do not specify the type of marriage in our qualitative instruments but allow respondents to interpret what they consider marriage to be.

Researchers in the United States and Malawi developed semi-structured interview and focus group discussion guides, with input from program staff in the Netherlands and Malawi. These guides were developed for this study and are provided as supplemental material (File: Supplemental 1 Qual Instruments). Guides were developed in English but interviews and focus groups were conducted in the local language (Chichewa, Yao, Tumbuka, and Tonga). The format of the focus group discussions and in-depth interviews was open-ended, facilitating a free flow of ideas from the respondents. Separate guides were designed for each respondent category with questions on topics related to marriage and the marital process, education, livelihoods, role models and adolescent social life including dating. Interviews and discussions were recorded and transcribed from the local language to English for analysis.

### Ethics

The study protocol was reviewed and approved by the Population Council Institutional Review Board in New York and the National Committee on Research in the Social Sciences and Humanities (NCRSH), an ethics committee established by the National Commission for Science and Technology (NCST) based in Lilongwe and authorized to provide ethics review and approval. Informed consent and parental permission (for unmarried minors) were acquired prior to participation in focus groups and interviews. We used female facilitators and interviewers with experience working with adolescents and training on addressing sensitive topics.

### Data analysis

Analysis of qualitative data included both thematic analysis of pre-determined topics but was also influenced by grounded theory [33], allowing for themes to emerge from the data. The research team used an iterative approach to developing the codebook including identifying relevant themes, developing an initial codebook, and refining themes as analysis progressed. Our coding procedure included first identifying text-based primary categories and subcategories, then grouping of the text-based categories into larger themes, and finally organizing themes into more abstract theoretical constructs. Two researchers (AM and NM) first reviewed a subset of transcripts and independently developed codebooks by annotating the transcripts. The researchers then met to discuss the codes, developing a refined codebook with parent and child codes, removing redundancies, and developing a preliminary hierarchy. The main themes that emerged from the data related to marriage and the marital process, premarital sex including transactional sex, livelihoods or lack thereof, the influence of poverty on the timing of marriage and sexual activity, and school drop out. After development of the codebook, a third reviewer was trained to apply the codes developed in the codebook and began coding the transcripts. Dedoose software (version 8) was used to establish intercoder reliability and for subsequent analysis [34]. Dedoose’s intercoder reliability test generated a Cohen’s Kappa coefficient of .77 across all codes and .81 on marriage-related codes. Two independent researchers (in the United States and Malawi) coded each transcript blinded to one another with memos used to record ongoing observations including reflections of how individual biases may influence interpretation of the results. Memos were reviewed by team members (AM and NM), some biases in interpretation were acknowledged and discussed (AM and NM) and some codes were refined to capture emerging themes. A reflexive statement (AM) is included as Supplemental 2 Reflexive Statement- AM.

After completion of coding, excerpts were examined for code co-occurrence, code occurrence by demographic characteristics, and code counts [35]. Code excerpts were exported to Microsoft Word for further organization around the larger themes or phenomena. We then applied corroborative counting of excerpts pertaining to marriage withdrawal by categorizing excerpts as positive, negative, or both after being exported in order to see whether there were sub-group differences in perspective [36].

## Results

Research participants were selected to ensure representation from married girls of the major ethnicities in the study area. As a result girls in the in-depth interviews were older than those in the FGDs (17.0 years versus 16.2) and more likely to report ever being married (42.8% vs. 22.6%). They were also not representative of girls in the baseline survey for this project [1] due to their older age and marital status. About one in four girls reported ever having a child (25.8% in FGDs compared to 28.5% in IDIs). Among parents in our FGDs, fathers were older than mothers (47.1 years compared to 39.8) and more likely to be currently married (100.0% compared to 76.0%). Tables 2 and 3 show participant demographics.

**Table 2:** Characteristics of Adolescents in FGDs and IDIs (*N* = 145)

	FGDs (***n*** = 124)	IDIs (***n*** = 21)	Baseline comparison (***n*** = 1020)
**Age (mean)**	16.2	17.0	14.9
**Ever Married**	22.6	42.8	15.9
**Enrolled in school**	54.8	33.3	64.0
**Has a child**	25.8	28.5	18.9

**Table 3:** Characteristics of Parents in FGDs (*N* = 47)

	Males (***n*** = 22)	Females (***n*** = 25)
**Age (mean)**	47.1	39.8
**Currently married**	100.0	76.0
**Number of children (mean)**	5.1	4.4
**Ethnicity**	68% Yao; 32% Tonga	64% Yao; 36% Tonga

### Laws and legal enforcement

We were interested to explore knowledge of minimum age at marriage laws in these communities. Understanding knowledge of minimum age at marriage laws is useful both due to the association between this research and the MTBA intervention but also to understand how knowledge of laws may influence behaviors related to those laws. We found that most respondents were aware of laws against child marriage; however, ability to identify the legal minimum age at marriage was mixed. Among fathers in a focus group in Mangochi, many reported that the legal age at marriage was over age 20. However, many others were able to correctly identify age 18 as the legal age at marriage. One focus group participant attempted to correct a fellow father who thought the legal age at marriage was 18:

*No, not 18 years, parents are the ones who say that the girl can get married at 18 but the government says the girl can get married at the age of 19 years and above.*

-Focus group of fathers, Nkhata Bay

We found that few participants believed the legal age at marriage to be under 18. When respondents were incorrect they often overshot the legal age at marriage by quite a few years.

#### Laws as a deterrent to child marriage

We were interested in understanding how knowledge of the minimum age at marriage law influences behavior and how enforcement of the age at marriage law is perceived in these communities. We found that the threat of consequences for breaking the age at marriage law is often mentioned by participants when asked about child marriage in their communities. Fathers in Nkhata Bay noted how fear of repercussions influences early marriage in their community:

*I: How common do you think early marriage [before 18] is?*

*P5: It is not common. People are afraid of paying penalties amounting to twenty five thousand kwacha [about 33 USD] to the village headman so they are not married below 18 years.*

*P4: The police unit frightens parents that when they accept early marriage they will be punished.*

-Focus group of fathers, Nkhata Bay

The fear of punishment includes fear that the bride and her parents could be punished as part of enforcement of minimum legal age at marriage laws. In addition to fines, often determined by community bylaws, punishment could also include arrest:

*Once we find a girl that is married, we terminate her marriage. Hence, most girls are scared to get married because they know that they will be arrested.*

-Focus group of mothers, Nkhata Bay

This fear of punishment as a deterrent was reported by both adults in the community as well as girls themselves. However, some girls believe that the way to reduce child marriage in their community is to leverage the illegality of the practice:

*[In addition to national law] chiefs too should come up with bylaws that any parent who found that their children are getting married should be fined large amounts of money so that everybody should fear that.*

-Focus group of unmarried girls, Mangochi

These two quotes also highlight an interesting tension we observed about the role of parents in child marriages. When discussing child marriage, parents in focus groups often blamed their children for the marriage, with daughters described as either being unruly and self-determined to be married or facing an almost inevitable union as a result of pregnancy. However, girls and key informants more often blamed parents for child marriages, suggesting that there is more parents should be doing to prevent these marriages from occurring.

#### Laws and legality driving marriages underground

Although the threat of punishment may prevent child marriages, an unintended consequence of minimum age at marriage laws and their enforcement is the hiding of marriages. Hiding marriages can happen both in terms of reporting, where parents and girls are less likely to report being married in surveys and censuses, as well as the literal hiding of marriage ceremonies by moving them out of view of the community. Mothers from Mangochi reported how some marriages now take place in secret due to fear of repercussions:

*Now for any early marriages that take place in this community the police definitely will come and arrest both the bride and groom and their parents too. They all get arrested by the police and there is a punishment fee that everybody has to pay. These early marriages are dissolved the same day by officials from [NGO name redacted] or child protection officers. So to avoid this embarrassment some think about doing it at night and secretly.*

-Focus group of mothers, Mangochi

Similarly, a female non-government organization (NGO) worker from Mangochi noted that despite the work of her organization and similar NGOs to prevent child marriages, theses marriages may still occur and be hidden. She feels that hiding marriages may be a direct response to NGO presence:

*The parents know that once the officials from [local NGO] know about this marriage it shall not be well received by them so they do it in secret.*

-In-depth interview with female NGO worker, Mangochi

Girls themselves also report that child marriages are being hidden. In response to whether child marriages occur in their community, married girls in a focus group from Mangochi discussed how it happens:

*P4: It is happening here, there is such kind of marriages happening.*

*P8: They are marrying them during the night.*

*P9: But when they are caught they are all taken to police and jailed there.*

*I : Why are they doing the wedding during the night?*

*P2: They are running away from being told that they are still young.*

*P7: They are doing that to make sure that the village head should not know what is happening there.*

*P8: When they have married one another they move out and stay in a different village. They are running away from being discovered.*

-Focus group with married girls, Mangochi

In extreme cases, girls may be sent to other villages or even countries where marriage laws may not be enforced. A primary school teacher from Nkhata Bay mentioned hearing about girls being sent away to marry as part of an arranged marriage.

*I: So you mean there are arranged marriages here?*

*R: Not around here but you find that the son of some other parents went somewhere for work maybe Tanzania or South Africa. Then you find that the parents of daughters may also send their daughters there so that they can get married while there so that there should not be any government policies to intervene.*

-In-depth interview with primary teacher, Nkhata Bay

### Marriage withdrawal as a strategy

Given the use of marriage withdrawal as a strategy to address child marriages [19], we asked participants about the practice of taking girls back from marital homes to their natal homes as a means to address child marriage. We explored both how marriage withdrawal works (*process)* as well as the effects of marriage withdrawal for the girl who is withdrawn (*outcome*). Responses from adolescent girls, parents of adolescents, and key community members, some of whom may be directly involved in the practice as part of their job or role in the community, offered subtle insights that we present here to help understand the widespread nature of this practice.

Many respondents viewed marriage withdrawal as a legitimate practice primarily because it has the support of community leaders and the authorities. Respondents suggest it is perceived as a necessary strategy when one learns of an early marriage within their community. In a focus group of married girls in Nkhata Bay we found that girls felt that early marriage is a problem that needs to be solved:

*P4: When a young girl is married, she must be withdrawn from marriage.*

*P6: The chief is helpful. He calls the community and addresses the problem, and the community discusses as one and they find a way of solving [the problem].*

-Focus group with married girls, Nkhata Bay

We were interested to hear about marriage withdrawal from law enforcement officials directly as they may be involved in the practice on the ground as part of child protection committees. In Nkhata Bay one male law enforcement official described the marriage withdrawal process:

*We first of all go to the girl’s parents, politely. After that, we go to where she has been married and we tell the husband that they have violated the rights of the girl. We tell them that the government discourages such marriages because the girls are still young and they have to complete their studies hence we are taking the girl to the police. [We tell the husband] “if you refuse, we will take you to the police instead of the girl”. This frightens them and then we take the girl and send her back to school.*

-In-depth interview with male law enforcement official, Nkhata Bay

When asked what happens if the parents refuse to agree to or participate in the withdrawal, he notes that law enforcement’s authority can be leveraged to make the withdrawal happen, but that they rarely have to enforce it with legal action.

*R: They cannot refuse because we have the authority granted to us by the government.*

*I: What authority?*

*R: That we should be looking after children in the village. We should also deal with thieves in the village and many other affairs of this village.*

*I: Have you ever taken someone to police because they refused to have their marriage terminated?*

*R: No. We have been handling such cases in collaboration with our village headmen and the people involved have been cooperative and understanding.*

-In-depth interview with male law enforcement official, Nkhata Bay

#### Different perspectives on advantages of withdrawal

Overall we found that most adults in the community see marriage withdrawal as positive and desirable for girls who are married as children. Marriage withdrawal allows girls to go back to school and can be viewed as ‘fixing’ the child marriage. As one father from Nkhata Bay put it, “*When the child has been withdrawn from marriage, she is advised that she is still young it is better for her to go back to school. So that you become a person who will be self reliant and you will be a role model who is educated.”* (Focus group with fathers, Nkhata Bay). This view of withdrawal as positive and curative for the problem of child marriage was also noticeable among key community members in our study. A male government official from Nkhata Bay recalls a withdrawal and how the NGO responsible helped that particular girl:

*R: I remember there is a family in this village, we told them that if they refuse to terminate their marriage, we would take them in police custody and they paid 6000.00 kwacha (8 USD) fine at the police station.*

*I: Is it parents that paid the fine?*

*R: Yes and the child went back to school. Therefore, it shows that the program has really helped in our village.*

-In-depth interview with male government official, Nkhata Bay

These adults report seeing themselves as responsible for the law and note that the consequences go beyond just punishing the older groom:

*According to the current laws regarding marriages and divorce, the recommended age for married is 18 years and older. If we discover that someone under age has been married whether willingly or forced, we do intervene and we make sure that the husband receives a serious punishment that sends a message across the community about the consequences of being involved in marrying young girls. We make sure that everyone that was involved in the arrangement of that marriage gets a punishment, even if a pastor was involved in officiating that marriage, he also has to receive a punishment.*

–In-depth interview with female social welfare officer, Nkhata Bay

Parents spoke of their responsibility and that of the authorities being inter-reliant in addressing child marriage. As one father in Mangochi put it, *“To end early marriage the parents and the village head should be working hand in hand”* (Focus group of fathers, Mangochi)*.* Mothers of adolescent girls in Mangochi note that mothers groups^3^ play an important role in bringing the cases of child marriage to the appropriate authorities for enforcement. In this case, mothers note that the child protection committee must be made aware of child marriages so they can act.

*In this community we also have mothers groups who play a big role in making sure that all girls are going to school. If they find out that a girl is married they follow her and they investigate. When they find out that it’s true that a girl is married, they report the matter to officials like the child protection committee. From there the police are informed.*

-Focus group with mothers, Mangochi

Yet, some adults acknowledged that the outcome of marriage withdrawal for girls is not always entirely positive. In a focus group of mothers of adolescent girls in Mangochi, respondents noted that girls may experience negative feedback:

*P5: The community doesn’t care about those that have been withdrawn from their marriages*

*P2: Others in the community mock them and they say “look at her she was suffering that’s why she has come back”*

*P4: Some in the community feel compassionate for them, they show their love to them and they advise them to think twice when they want to get married again.*

-Mothers of adolescent girls, Mangochi

In this same group, the mothers all noted that the community does not provide any support to girls who are withdrawn.

In contrast to many of the positive perceptions of marriage withdrawal by adults in the community, we found that girls have slightly different perspectives on marriage withdrawal. Some view it positively, relating stories of girls whose lives were improved. In Table 4 we examined excerpts regarding outcomes for girls who are withdrawn (*n* = 76 excerpts) and find that more than half of excerpts from adults mention positive-only outcomes for girls who are withdrawn (52.2%), compared to 28.3% of excerpts from adolescents. More adolescent girls mentioned negative-only outcomes (47.1%) than adults (30.4%) while those reporting both positive and negative outcomes are similar (28.3% among adults; 24.5% among adolescents).

**Table 4:** Perception of Marriage Withdrawal Outcomes (*n* = 76 excerpts)

	Outcome for girls who are withdrawn			
Excerpt participant type	Positive only N (%)	Negative only N (%)	Positive and Negative N(%)	Total
Adults	12 (52.2)	7 (30.4)	4 (17.4)	23
Adolescents	15 (28.3)	25 (47.2)	13 (24.5)	53
Total	27	32	17	76

Among the positive perceptions of outcomes for girls, married girls in a focus group in Nkhata Bay noted that a girl who is withdrawn has the ability to go back to school:

*They go to the man who has married the young girl, they withdraw the young girl from marriage. This is how early marriages end. Even if the young girl is pregnant, the girl will take care of the pregnancy at her parents’ house and the man will be single. After giving birth, if she is still willing to go back to school and has funds to pay the school fees, then she goes back to school.*

-Focus group with married girls, Nkhata Bay

Yet other girls focused on the negatives associated with marriage withdrawal, both for the girl as well as potentially for her children:

*Her life looks so miserable and she will never think of getting married again. Most of the time children are the ones who suffer most when the man and woman divorce because these children are innocent and they don’t have any one to take care of them.*

-In-depth interview with unmarried girl, Mangochi

Another adolescent girl from Mangochi suggested that withdrawn girls may have so few options that prostitution may be a means to get money:

*I: What are her options once back in her community?*

* P: She thinks of doing prostitution.*

*I: Anything else?*

*P: Nothing.*

*I: Why does she choose prostitution?*

*P: So that she can get money.*

-In-depth interview with unmarried adolescent girl, Mangochi

Some girls noted that the consequences of marriage withdrawal could be both positive and negative within the same community:

*Some go back to school again, others start small businesses like selling doughnuts, while others start to look for work. When a girl comes back to her parents’ home people in the community start to mock her, while others encourage her to go back to school.*

-In-depth interview with unmarried girl, Mangochi

Responses indicate there may be wide ranging options for girls who are withdrawn, from going back to school to more risky endeavors to address economic needs. Adolescent girls also noted that marriage withdrawal doesn’t always work as intended. Unmarried girls in Nkhata Bay noted that sometimes girls will still go back to their marriage after being withdrawn:

*P8: There are some young girls who are married, and when the organization wants to withdraw her from marriage, she refuses.*

*I: They refuse to be withdrawn from marriage?*

*P8: Yes, they refuse to be withdrawn from marriage.*

*P6: Some young girls are withdrawn from a marriage and after reaching their home they go back to their marriage.*

-Focus group discussion, unmarried girls, Nkhata Bay

## Discussion

Our findings suggest that there is considerable knowledge about the minimum age at marriage law in these communities. However, we also find that there are ways around the law in situations where girls or their parents want them to get married. In cases where the age at marriage law is community- or parent- enforced, we found that marriage withdrawal is a generally accepted process by which girls may be taken back from marriage to their natal home. However, we also find mixed feelings about the advantages to marriage withdrawal, with adults in the community generally holding more positive views of the procedure compared to adolescents. We also find tension between who is considered ‘at fault’ for a child marriage, with adults more often placing the blame on girls for the marriage or the pregnancy necessitating the marriage while girls suggest that parents should play a bigger role in enforcing laws. This tension of where the responsibility to prevent child marriages is placed may be resolved in part by marriage withdrawals. Withdrawals may allow for parents to take a socially accepted stance of participating in the withdrawal and breaking up the marriage, responding to the problem of child marriage in a curative way. As we heard from parents in focus groups, generally they feel that they are not able to prevent child marriages from occurring. Withdrawals may also allow for atoning for a child marriage without paying associated fines.

We found that the opportunities for girls who have been withdrawn are to attend school, become involved in some income-generating activities (legal or illicit), and get married again at a later age. These opportunities are limited in the same way they are limited for girls who are not withdrawn. Opportunities to earn money are likely dependent on others offering girls piecemeal work. Some girls are fortunate to have parents or other relatives who support them. Still some participants mentioned girls getting involved in prostitution in order to have some money. For girls who are withdrawn, the likelihood of successfully navigating economic challenges to support themselves (and their offspring) is low in a context where never-married girls also face an economic situation that limits their earning potential [37, 38]. This suggests that if marriage withdrawals continue to occur as part of a broader child marriage strategy within communities, those conducting withdrawals should make sure that communities are prepared to offer girls assistance with education re-entry (e.g., school fees) or transitions to livelihood opportunities (vocational training or seed grants) to help ensure a successful transition post-withdrawal.

Beyond education and livelihood opportunities, this transition post-withdrawal is also important socially. We found that most adolescent respondents noted the social stigma associated with marriage withdrawal. Stigma associated with marriage withdrawal may have important consequences, especially for girls who find they cannot go back to school or those who are already pregnant. However, we did not find stigma mentioned among adults interviewed as part of this project suggesting that adolescents may be better attuned to the difficulties withdrawn girls face. The lack of mention of stigma amount adults in our sample could perhaps be due to the adults included in the study being either parents themselves or affiliated with the child protection sphere through working with an NGO or local government.

There are some limitations that should be considered in interpreting these findings. Adults included as key informants in this research were selected either for their position (e.g., government leader; teacher) or because they were referred to the research team by local program contacts. Due to their roles or affiliations, they may have particular views on laws and legal enforcement that are not shared by other adults in the community. Similarly, some of this research was conducted in program implementation areas where local NGOs have been active. We view this as both a strength and limitation: a strength is that we can examine perceptions of enforcement where we know practices like marriage withdrawal occur. However, a limitation is that these communities may have close ties to the local NGOs and may be different from communities where NGOs have limited influence. As the MTBA approach includes working with child protection communities, these areas may be less representative of other rural areas in Malawi. We also note that the ‘quantification’ of qualitative data such as in Table 4 can be problematic and these data may not accurately assess individual perceptions of marriage withdrawal. However, in the process of qualitative data analysis multiple authors (AM, NM) noted that girls appeared to view marriage withdrawal in a slightly different way and we wanted to explore that more fully. Future research may examine this using other methodologies to more fully understand the nuances in perspectives on marriage withdrawal and its outcomes.

In recent years there has been a global discussion facilitated by organizations like Girls Not Brides about the importance of enacting and enforcing age at marriage laws to combat child marriage. Although we agree that laws against child marriage send an important message that children under age 18 should not be married, we also argue that legal approaches by themselves are insufficient for reducing child marriage in most contexts and in some cases have unintended consequences. For example, research in India suggests that enforcement of child marriage laws has a negative effect on girls’ agency and is used to punish girls for exercising choice in whom to marry [39]. We found that the minimum age at marriage law was successful in that some participants reported fear of repercussions, both legal and social, to early marriage and this likely prevented some child marriages from occurring. However, others noted that the law encouraged the hiding of child marriages instead. When child marriages are driven underground this obscures the true toll of child marriage in these communities and may reduce resources available to address child marriage.

The use of marriage withdrawal as a strategy to address child marriage sends the message that marriage before age 18 is illegal and will not be tolerated within that community. However, we also note that marriage withdrawal is a curative approach that does little to prevent child marriage in the first place. Withdrawal also fails to address the primary drivers of child marriage that may be more influential in determining when a girl is married. This research suggests that despite laws and legal enforcement, marriage before age 18 still occurs and that to effectively target early marriage in these communities we need to better address more direct causes of child marriage in Malawi such as pregnancy and poverty, including a lack of work opportunities for girls. A complementary analysis of these data suggest that addressing pregnancy as a primary factor driving child marriage may be a more effective strategy for combating early marriage in Malawi (findings presented elsewhere). These findings have implications beyond Malawi and suggest that legal enforcement strategies need to complement other approaches that address more proximal causes of marriage, such as a pregnancy or poverty and financial insecurity.

## Conclusions

Despite the recent passage of the minimum age at marriage law in Malawi we find that in some rural communities the legal age at marriage is still not widely known and child marriages are still occurring, sometimes in secret. We also find that despite media stories and NGO messaging about marriage withdrawal as a successful tool to combat child marriage, this practice is viewed more positively by adults in the community while girls report more skepticism about whether this practice leads to better outcomes for girls. This research suggests that knowledge and enforcement of minimum age at marriage laws as a strategy to prevent child marriage should be complemented with other approaches that directly address drivers of child marriage in that context. This research also suggests that if marriage withdrawals continue to occur as part of a broader child marriage strategy, communities and governments should consider other supports withdrawn girls need after returning to their natal homes.

## Supplementary Information

**Additional file 1.** Malawi Qualitative Instruments. This file includes the qualitative instruments developed for and used in conducting qualitative focus groups and in-depth interviews for this research.

**Additional file 2.** Reflexive Statement- Andrea J. Melnikas, MPH, DrPH. This file includes a reflexive statement from the first author.

## Data Availability

The qualitative data were collected in rural areas and included sampling adolescent girls married before age 18 and discussing child marriage, both of which are highly sensitive. De-identification and posting to a repository while maintaining the content is challenging given those sensitivities. However, qualitative data may be made available upon reasonable request to the corresponding author.

## Abbreviations

ACRWC: African Charter on the Rights and Welfare of the Child
DHS: Demographics and Health Surveys
IDI: In-depth interviews
IRB: Institutional Review Board
FGD: Focus group discussion
MTBA: More Than Brides Alliance
NCRSH: National Committee on Research in the Social Sciences and Humanities
NGO: Non-governmental organization

## Footnotes

1 MTBA is a holistic program to address early marriage through multiple strategies, including: empowering girls through life skills, access to sexual and reproductive health services, livelihoods, education, community engagement to address social norms, and interventions to create a favorable legal and policy environment. The program is currently implemented in India, Malawi, Mali, Niger, and Pakistan and implementing partners include Save the Children Netherlands, Oxfam-Novib, Simavi, and each organization’s local partners. More information is at morethanbrides.org

2 Child protection committees are groups of community members as well as law enforcement members. They are typically government affiliated but may also receive support and training from NGOs. In the MTBA program, local partners work to strengthen child protection committees in the communities where they work.

3 Mother groups are a government initiative to provide girls a social support system apart from their families. These are available in every school as a policy.
