# Supplementary material for: Perceptions of minimum age at marriage laws and their enforcement: qualitative evidence from Malawi
Source: BMC Public Health. 2021 Jul 8;21:1350. doi: 10.1186/s12889-021-11434-z (PMC8268505; doi:10.1186/s12889-021-11434-z)
Supplement: Supplementary file 1 — Additional file 1. Malawi Qualitative Instruments. This file includes the qualitative instruments developed for and used in conducting qualitative focus groups and in-depth interviews for this research. [file 12889_2021_11434_MOESM1_ESM.docx]

**Study Instruments -- Malawi**

- Focus group discussion guide for parents of adolescents ages 12-19
- Adolescent focus group discussion guide for girls ages 12-19
  - unmarried
  - married
- In-depth interview guide for girls ages 15-19
- In-depth interviews guide for key influential age 18+

**Focus Group Guide for Parents of Adolescent Girls**

| Study ID | Age | Marital status | Age at marriage | Number of children | Years of education | Ethnicity |
| --- | --- | --- | --- | --- | --- | --- |
|  |  |  |  |  |  |  |
|  |  |  |  |  |  |  |
|  |  |  |  |  |  |  |
|  |  |  |  |  |  |  |

**Definition of marriage and perceived readiness for it**

1. In your opinion, what is the ideal age for a girl to marry? Why? What are the signs that she’s ready to get married?
   1. PROBE for what age is considered an early marriage and how it is defined (i.e. tradition, ready to have kids, physical or psychological maturity, done with schooling, ready for household chores, legal age)
2. Is it desirable for a woman and her husband to be the same age and have the same level of education? Why or why not? Are there any problems when there is a big age difference?
3. What are the motivations for marriage in your community?
   1. PROBE: fear of pregnancy, kinship, economic gains
4. What is the legal age at marriage for girls? How does the law influence marital age in your community?
   1. PROBE: what happens in reality versus ‘on paper’?
5. Do authorities ever get involved in marriages of young girls? How does that happen and what do the authorities do?
6. If early marriage was an issue in your community, what might your community do to address this? Who is responsible for addressing it? What structures are available to address this? Does your community have NGOs, community groups or child protection structures?

**Marital transactions**

1. For the majority of people in this community, what is the process that precedes marriage? Who decides whether a girl will marry? Are there different rituals for different ethnicities?
   1. PROBE: girls’ agency, duration of negotiation, stages, cohabitation, contact between the girl and her potential husband/in-laws
2. Who are the people who are involved in the marital process for girls and what are their motivations?
   1. PROBE: Decision makers, negotiators, role for each of them; advantages they obtain from organizing marriages? And for families what is the role of kinship in motivating the decision to marry girls?
3. Who decides the exchange of gifts/money between the families and how does that influence the duration of time before the marriage?
   1. PROBE: type of gifts, sum of money, sociocultural rituals
4. Are there situations where a planned marriage does not happen? Why?
   1. PROBE: specific circumstances
5. Why do marriages end in this community? How common is divorce? What about girls being withdrawn from marriages. How and why does that happen?
   1. PROBE: status of women/girls after marriage fails; opportunities for remarriage

**Alternative Pathways**

1. How far do girls typically go to school in your communities? What typically precedes the end of formal schooling for girls in your communities?
   1. PROBE: decision-makers in drop out process; “finality” of drop out and chance of return
2. What do girls do when they are no longer in school?
   1. PROBE: (how) does this differ for boys?
3. What are the options for girls who do not get married?
   1. PROBE: work opportunities; staying in school
4. Who are role models for adolescent girls in your community?

**Initiation Ceremonies**

1. How common are initiation ceremonies in your community?
   1. PROBE: differences for boys and girls; ethnic group differences
2. What role do initiation ceremonies play in marking a transition to adulthood and readiness for marriage?
3. What are the benefits for girls going through initiation ceremonies? Are there are any detriments?
   1. PROBE for detail, examples

**Closing**

1. Is there anything else you think we should know about early marriage in your community?

**Focus Group Guide – Female Adolescents**

| Study ID | Age | Marital Status | Enrolled in school? | Has a child? | Ethnicity |
| --- | --- | --- | --- | --- | --- |
|  |  |  |  |  |  |
|  |  |  |  |  |  |
|  |  |  |  |  |  |
|  |  |  |  |  |  |

**Typical trajectories for girls schooling and mobility**

1. Imagine a typical girl in this village. If she is in school, at what age does she finish her schooling? Why? What does she do afterwards? How is she different from a girl who has never been to school?
   1. PROBE: domestic chores, work, non-formal education, marital plans, livelihoods
2. When do girls in your communities stop going to school? What are the reasons for no longer going to school?
   1. PROBE: relationships, pregnancy, financial barriers, negative experience at school
3. Do girls who drop out of school ever go back? What does that look like?
4. For the typical girl, describe her mobility in the community, where does she go regularly and with whom? How does her mobility change throughout adolescence (meaning 10-19 years old) and why? What are her social networks?
   1. PROBE: permission to go out, from whom, the age at which mobility changes and why, friends, sources of information [for married girls only: how does mobility change for girls after marriage?]
5. For a typical girl in your community, what is her social life like? When does she start dating? What is the ‘right’ age to start dating?
6. What does dating look like in your community? Are there different ‘types’ of men/boys that girls in your community date? Is one ‘type’ better or worse than another?
7. Where does a girl in your community go when she needs health services? What about when she wants to use contraception? How easy is it for a girl to access sexual and reproductive health services?
   1. PROBE: parental involvement, black market exchange, money for services, youth-friendly services, quality of services

**Motivations for marriage**

1. What are the reasons girls in your community get married? Which are the most important and why?
   1. PROBE: (lack of) opportunities for girls, money/economic gains, kinship, fear of girl becoming pregnant
2. What say do girls in your community have about whom to marry and when?
   1. PROBE: does/how does level of agency vary among girls

**Post-Schooling & Livelihoods**

1. What are the opportunities for girls once schooling ends?
2. What types of jobs do women in your community do? What about non-formal ways of earning income?
3. Does technology play a role in jobs in your community? What about in your daily life?
4. When a girl or young woman makes money, what does she do with that money? Where can she save? What is she saving for?

**Alternative Pathways**

1. Think of a woman in your community you admire or consider a role model. What does her life look like? Why do you admire her? How did she get to where she is?
   1. PROBE: special circumstances/advantages, ‘alternate’ pathway

**Community**

1. How does your community come together to address problems? What role do adolescent girls play in that? What role should they play in your opinion?
2. Who are the people with the most important opinions in your community? Why are their opinions the most important? Do you agree with them being most important?
3. Do you have any examples of how your community came together to address an issue, either big or small? How do you think it was handled?
4. Do you think your community should do something to address early marriage? Or if they are already doing something to address early marriage, do you think they are doing the right thing? Will it help? How will you know it was effective?

**In-Depth Interviews with Adolescent Females**

Participant ID: _________________

Age: _________________

Marital Status:__________________

Age at marriage:________________

Number of children: _____________

School status: __________________

**Schooling**

1. Tell me about your experience with school. Did you ever attend? (If not skip to question 4)
2. Are you currently enrolled? If so in which type of school and at what level?
   1. PROBE: Primary, secondary, passed exams.

If not currently enrolled

1. Why are you not currently enrolled? (Skip to number 5)
   1. PROBE: principal reasons, cost, other obligations, attitudes towards girls schooling, has a child

If she never went to school)

1. What were the primary reasons why you didn’t attend school?
   1. PROBE: cost, chores at home, family attitudes toward girls’ schooling, siblings’ schooling

**Living arrangements**

1. What is your living arrangement-whom do you live with and how long have you lived there?
2. Where did you live before? (if reported that didn’t always live at current residence)
   1. PROBE: location of previous residence, distance from current, migration, etc.

**Employment**

1. Tell me about employment opportunities that you currently have or have had in the past. What was the type of work? How did you find it? Was it at home in the village or did you have to move elsewhere for this opportunity?
   1. PROBE: informal and formal

Read next questions if yes she has worked/is working; if not skip to 12)

1. How long have you been/were you engaged in this job?
   1. PROBE: age when this job started and now
2. What were/are some of the positive things about this type of job for you?
   1. PROBE: skills learned, pay (how much), working conditions
3. What were/are some of the challenges about this type of job?

PROBE: skills needed, pay (how much), working conditions

1. Have you used/Do you use specific skills to help you better do this job?

PROBE: job-specific skills (i.e. cooking, cleaning) or transferrable skills (numeracy, negotiation)

1. What did you do/do you plan to do with the money earned in this job? Do have specific goals in mind?

PROBE: sending to family, trousseau, short and long-term savings goals, where does she keep her money

1. What kind of support do girls like you need to save their money?

PROBE: knowledge of savings, a safe place to save their money (where)

**Marriage and childbearing**

1. Have you ever been engaged or married?
2. Are you currently engaged or married?
3. How old were you when you got married?
4. Did you live with your husband before marriage?
5. Who was part of the decision that you would marry, and what were the main reasons?
   1. PROBE: parents, girls herself, (lack of) opportunities for girls, money and economic gains, kinship, fear of girls becoming pregnant (or being pregnant)
6. How was it decided whom you would marry, and what type of union was it/will it be?
   1. PROBE: free choice or arranged between families, polygamous/monogamous, civil vs. religious vs. traditional
7. Can you describe what happened between those initial discussions about marriage and now? How long has the process been, and what have been some of the key stages in it?
   1. PROBE: gift giving between families, lobola, cohabitation, consummation of marriage
8. Have the discussions about having a child begun? If so, are you pregnant or when was the child born in relation to the marriage?
   1. PROBE: who wanted her to get pregnant; duration between cohabitation and pregnancy or ceremony and pregnancy; (If relevant) How many children do you have?
9. Please describe the happy events in your marriage
   1. PROBE: love for partner, status associated with marriage, birth of a child and her age at the time of becoming a mother, harmony with in-laws
10. Please describe the difficulties encountered in your marriage. What happened? How did you resolve these difficulties?
    1. PROBE: Problems with husband, in-laws, conflict with people in her life
11. What advice do you have for girls who will be marrying soon?
    1. PROBE: information that she needs, skills that are necessary and who can teach them to her

**Marriage Withdrawal**

1. Have you heard of girls being withdrawn from marriage?
2. What have you heard about the reasons girls get withdrawn from marriages?
   1. PROBE: circumstances she has heard about, age of that girl
3. How common is divorce in your community? Who initiates the divorce? What are the reasons for divorce?
4. What happens to a girl after she is withdrawn from a marriage or divorced? What does her life look like?
   1. PROBE: what about her children?

**Interventions**

1. Have you seen any activities in your community that you thought were helping adolescent girls?
   1. PROBE: how they were helpful; who attended;

2. What can programs in your community do to support girls at risk of early marriage?

**In-depth Interview Guide (key influentials)**

Name: __________________

Age: ___________________

Education: _______________

Background: (economic background, how long living in this village): ____________________

How long have you been doing this work? (for professionals): _________________________

**Education:**

1. How do you feel about the educational opportunities for girls in your community? Do most adolescent girls go to school? What if any might be hindering their schooling?
2. Do girls and boys have equal opportunities? Do you think that girls shouldn’t go to school after a certain point? What are your thoughts about girls education?
   1. PROBE: girls in school after marriage, birth
3. How are the schools in your community? Are facilities adequate and accessible? What can be done to improve facilities?

**Livelihoods and Work**

1. What is your impression about economic situation and work opportunities for adolescents and young people in the community? What are the main sources of livelihood for young people in the community?
2. What are the work opportunities for women? Are women in your community earning money and are they financially literate? Are they parts of savings and loans groups? Are there opportunities for entrepreneurship for women?

**Marriage:**

1. What are the traditions surrounding marriage in your community? Do girls get married early? Are there more love marriages or are marriages still arranged? Are girls often taken out of school to get them married off? Why do you think this happens?
2. Do the authorities ever intervene when a girl gets married early? How does that process happen in your community?
3. What are the perceptions of the “child protection” groups in your community?
4. At the time of marriage, do families give each other money and/or gifts? Is there a culture of Lobola in this community? How does Lobola influence marriages in your community? Are there specific guidelines or traditions around Lobola in your community?

**General and Reproductive Health:**

1. Do you think health services for the general population are adequate in your community? What about reproductive health services for women and girls?
2. Do young people, especially girls, have access to reproductive health services when they need it?
3. Do young people in your community receive good education on sexuality and things like puberty and menstruation and family planning and contraceptives from a young age? Do you think what is being offered is adequate? What should change?
   1. PROBE: where do they receive it (initiation ceremonies? Family? Community?)
4. Do you think young people in your community are aware of STIs and HIV and how it is transmitted?

**Migration:**

1. Is there a lot of migration from the community or do most young people stay? If people migrate, what are they key reasons that they migrate? How does migration affect girls?
   1. PROBE: marriage, economic opportunities, climate
2. Do young people who migrate send back money to their households? Is that a useful thing? How often do migrants return?

**Vulnerability:**

1. What about their vulnerability? Are there specific social risks that young people, especially girls in the community face?
2. Are young girls discriminated against in your community or harassed? If yes, in what ways? Is it generally safe for girls in the community to move around?
   1. PROBE: Where is it not safe? Why?
3. What resources does the community offer to young people to cope with financial needs?
4. Do you hear of girls and women facing problems such as gender based violence in your community? If yes, what do you think is the cause of the problem? What can be done to combat it?
5. Are youth involved in bad behaviors like using drugs, drinking alcohol or smoking? How do you think this can be solved?

**Gendered Norms**

1. What are some important gendered norms and practices that make boys and girls men and women different from each other?
2. What are some important norms/ institutions/ community characteristics that promote or hamper girl’s and women’s work, marriage, mobility, what women can do, where women can work?
3. Are there still some harmful practices that are still practiced as tradition in your community? If they are, what are they (e.g. initiation rites)? Do you think it is right to continue these traditions?

**Youth Culture**:

1. How do you feel about the state of youth culture today? Do youth use a lot of technology like mobile phones and computers?
2. Are young people dating more freely? Do you think sexual activity is happening earlier than normal and before marriage? What are the norms and what is considered deviant behavior in your community?

**Perceptions about NGOs and Government and their work in development of the community:**

1. What is the perception of the community and their opinions regarding the NGOs working in the area? How are NGOs perceived relative to government institutions? How are their perceptions of the government?
2. What is the perception of the MTBA program? What activities have you observed?
