# Supplementary material for: Perceptions of minimum age at marriage laws and their enforcement: qualitative evidence from Malawi
Source: BMC Public Health. 2021 Jul 8;21:1350. doi: 10.1186/s12889-021-11434-z (PMC8268505; doi:10.1186/s12889-021-11434-z)
Supplement: Supplementary file 2 — Additional file 2. Reflexive Statement- Andrea J. Melnikas, MPH, DrPH. This file includes a reflexive statement from the first author. [file 12889_2021_11434_MOESM2_ESM.docx]

**Reflexive Statement- Andrea J. Melnikas, MPH, DrPH**

I am an adolescent health researcher trained in public health in the US and living in the US (outsider position). My research on adolescents has focused on prevention of risky health behaviors and this prevention lens influences how I view adolescent choices with regards to health, education, and family formation. My research training included an emphasis on grounded theory approach, specifically learning this approach using a large qualitative study that involved family formation and transition to adulthood among young adults in an urban center in the US.

Because of my outsider position, I relied on guidance from my colleagues NM, a female Malawian researcher trained in qualitative methods and extensive experience working with adolescents, and JM, a male Malawian research director with expertise in developing and conducting research on adolescents in Malawi. Notably NM and JM are not from the communities where the research occurred but have experience working in these areas. We worked with More than Brides Alliance program partners in these communities to review the instruments and workshop insights and results during analysis.

This manuscript represents a component of a larger research study, the More than Brides Alliance, of which I served as project director and investigator and SA serves as principal investigator. That project includes research in four other countries with a burden of child marriage. This influences how both I and SA think about child marriage and adolescent health more broadly because we often apply a compare/contrast lens between settings. For example, child marriage research in Malawi represents a less studied topic than in India (part of this project) or Bangladesh (part of a larger body of my work), settings where the drivers and context are different. As a less studied context I need to ask myself during analysis if the insights and findings in the qualitative data are influenced by what I know about other contexts.

The qualitative research in this study took place after adolescent surveys and in my view were an opportunity to better understand the lived experience of girls in these communities so that programs to serve these girls may be better developed to meet their needs. I believe in centering girls in these discussions and have tried with my partners to capture their experiences and reflect on how those may differ from what we find quantitatively or may differ from what we hear from adults in these same communities.
